# Supplementary figures and images for: Chickpea NCR13 disulfide cross-linking variants exhibit profound differences in antifungal activity and modes of action
Source: PLoS Pathog. 2024 Dec 2;20(12):e1012745. doi: 10.1371/journal.ppat.1012745 (PMC11637438; doi:10.1371/journal.ppat.1012745)

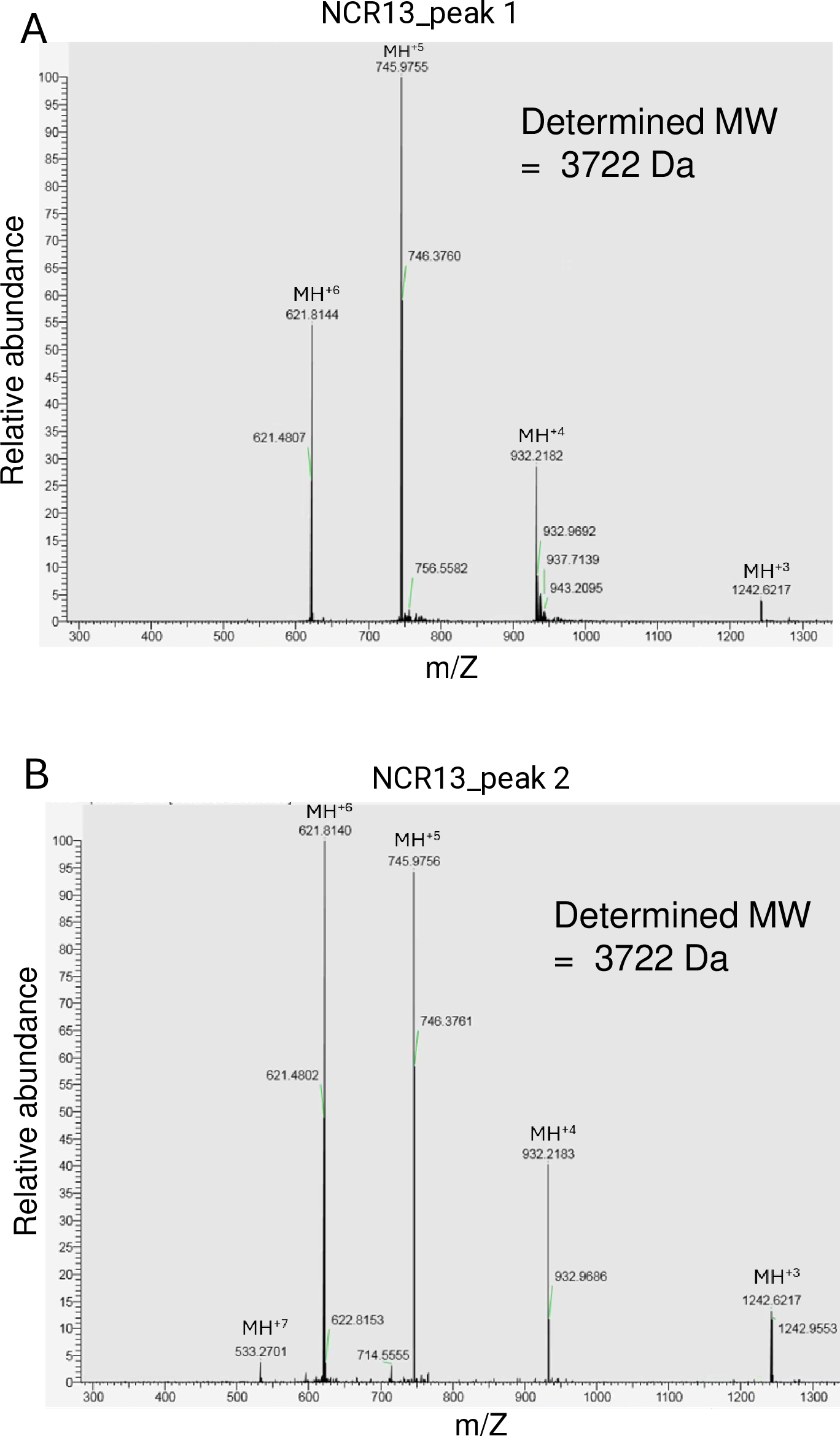

Supplement: S1 Fig — (A-B) The mass spectra of NCR13_peak 1 and NCR13_peak 2 shows major peaks corresponding to different charge states of the peptide. MW = Molecular weight. (TIF) [file ppat.1012745.s001.tif]

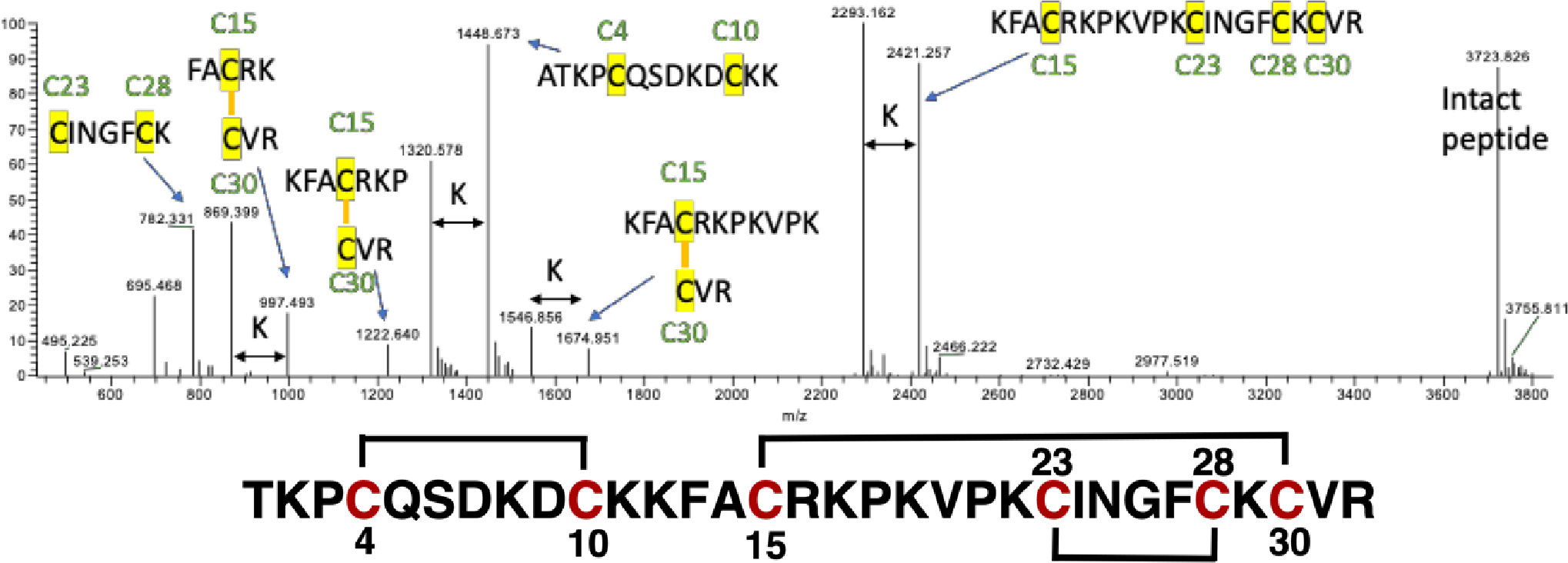

Supplement: S2 Fig — The primary amino sequence of NCR13 contains six cysteine residues which means there are 15 possible combinations of disulfide bond formation. In nature, each NCR is expected to fold with one specific disulfide bond combination. High-resolution mass spectrometry on a trypsin digested sample unambiguously shows the pattern is C4-C10, C15-C30, and C23-C28 for NCR13_PFV2 as illustrated in the analysis above. There are at least three major digestion products that correspond to a C15-C30 disulfide and two corresponding to a C23-C28 disulfide. This information was crucial in solving the NMR solution structure for NCR13_PFV2. (TIF) [file ppat.1012745.s002.tif]

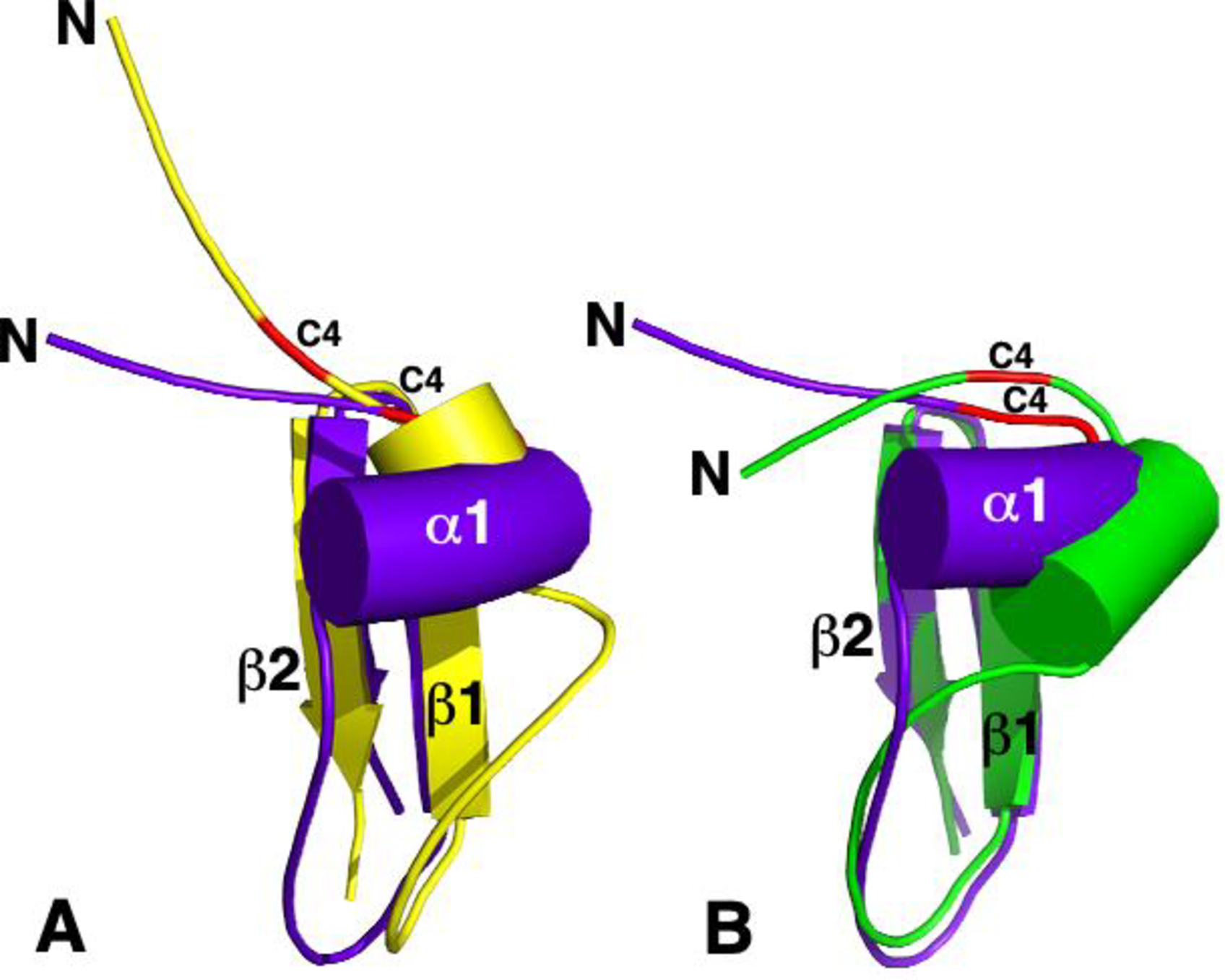

Supplement: S3 Fig — Cartoon representation of the structures of NCR13_PFV1 (purple) and NCR13_PFV2 (grey) superimposed on the β-sheet (V20 –V31). Helices are illustrated as cylinders. In NCR13_PFV2, the helix sits off to the side of the β-sheet while in NCR13_PFV1, it sits over the top of the β-sheet’s face. This results in a slightly more compact structure for NCR13_PFV1. (TIF) [file ppat.1012745.s003.tif]

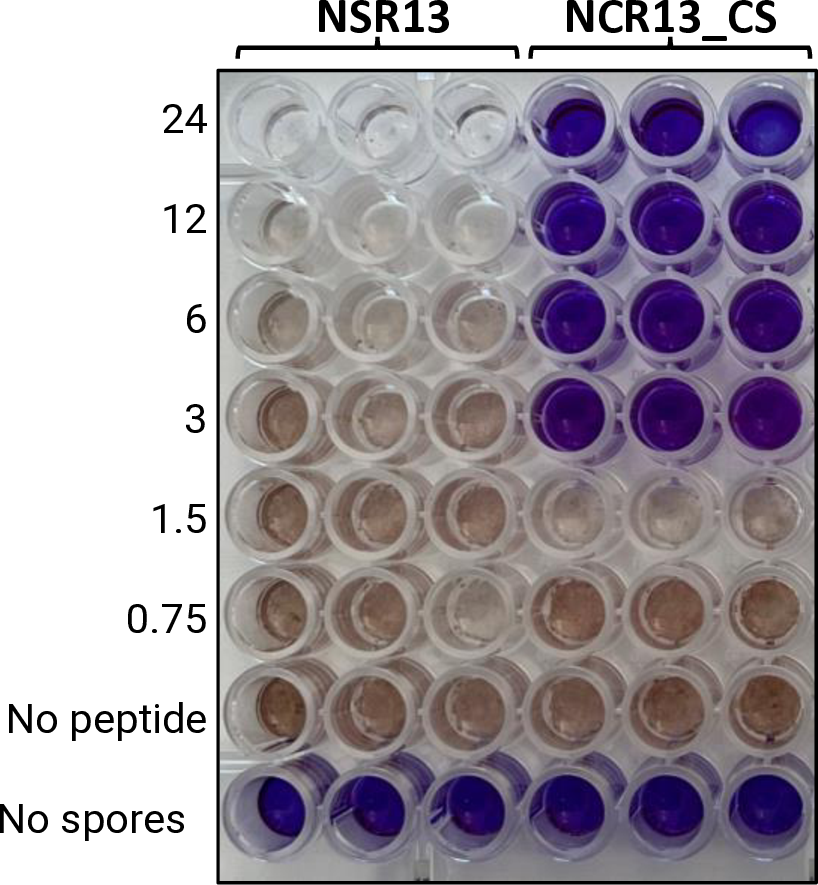

Supplement: S5 Fig — Comparison of the antifungal activity of a full disulfide knockout, NSR13, and chemically synthesized NCR13 (NCR13_CS) Fungal cell viability assay performed with resazurin. A change from blue to pink/colorless signals resazurin reduction and indicates metabolically active B. cinerea germlings after 60 h. (TIF) [file ppat.1012745.s005.tif]

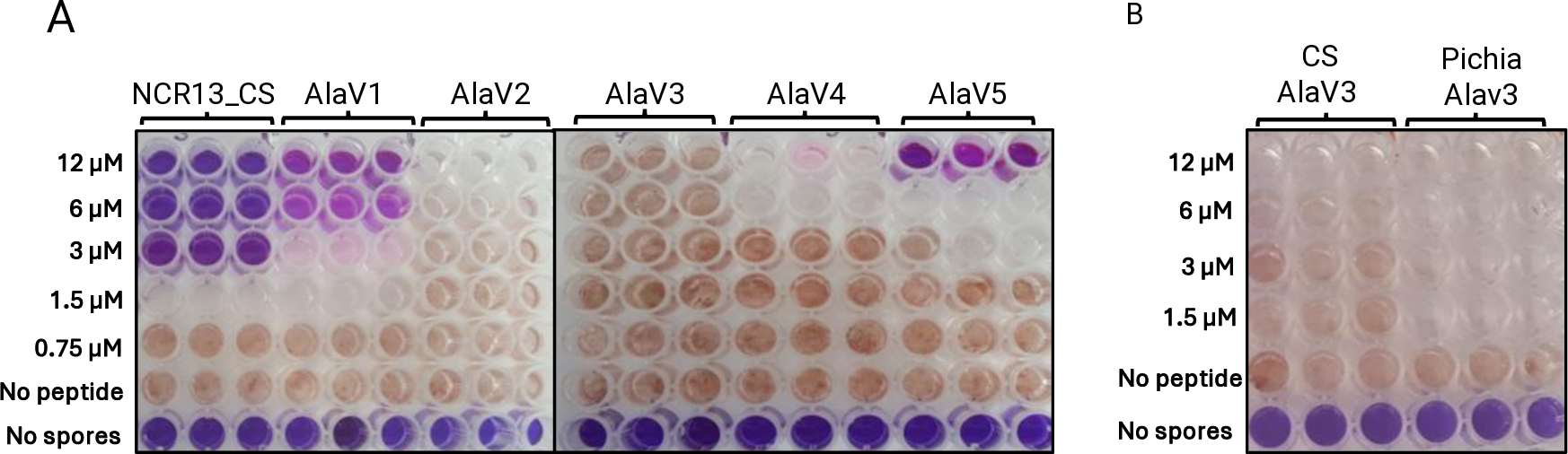

Supplement: S6 Fig — (A) Antifungal activity of synthetic NCR13 and NCR13 alanine mutant variants. The latter constructs were generated by substituting a window of alanine residues within the NCR13 core sequence, leading to the creation of constructs NCR13_AlaV1 through V5. (B) Comparison of the antifungal activity of chemically synthesized NCR13_AlaV3 and P. pastoris produced NCR13_AlaV3. All experiments were performed against B. cinerea using the resazurin fungal cell viability assay. A color change from blue to pink/colorless signals resazurin reduction indicating metabolically active fungal spores after 48 h. For each concentration of peptide, three biological replicates were used. Calculated MIC values are provided on the side of each experiment. (TIF) [file ppat.1012745.s006.tif]

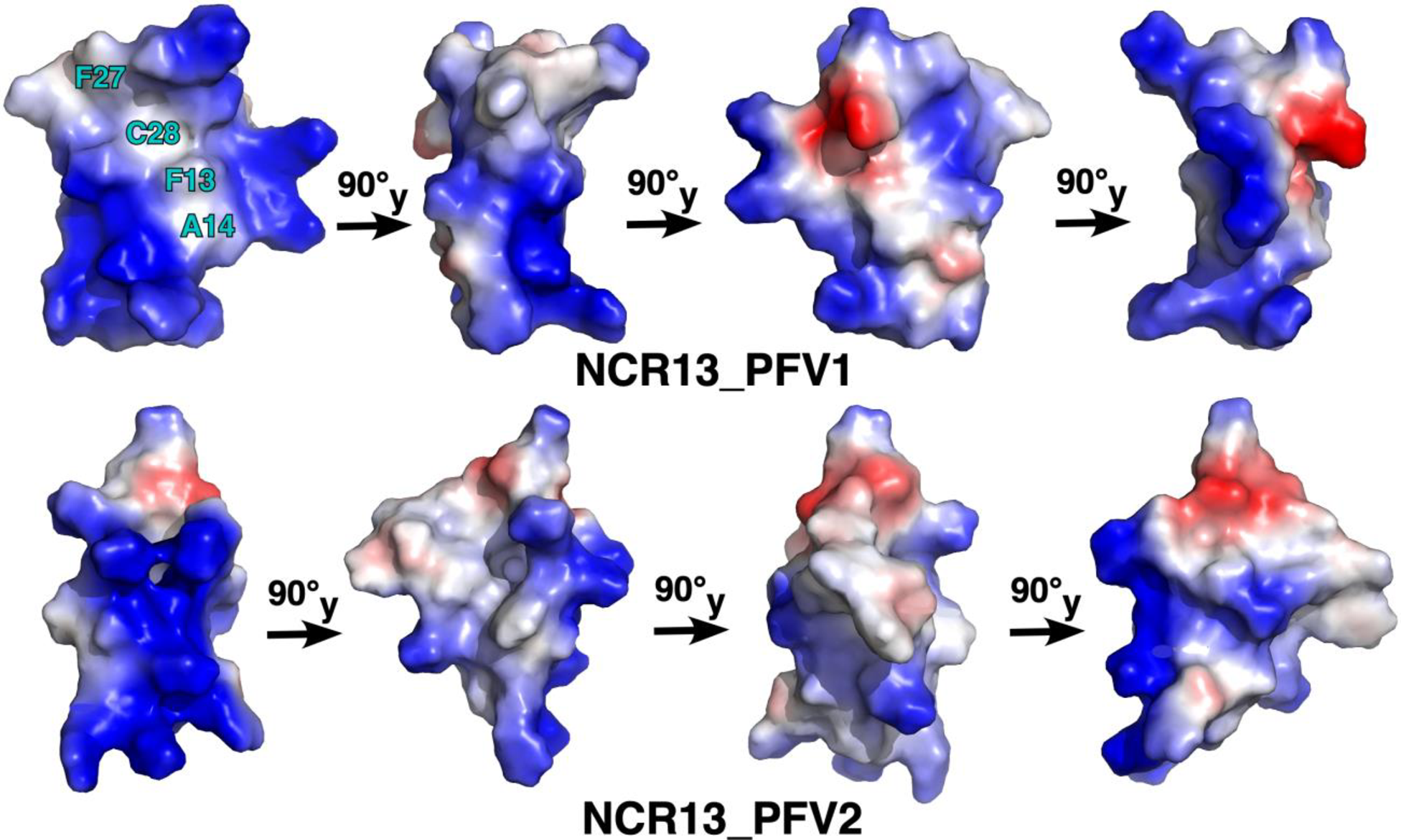

Supplement: S7 Fig — Electrostatic potentials at the solvent-accessible surface of NCR13_PFV1 and NCR13_PFV2 with negative regions colored red and positive regions colored blue. All four faces of the protein are shown by rotating the first structure ~90° around the y-axis (counterclockwise looking down from the top of the y-axis). Each peptide contains ten positively charged and two negatively charged side chains that are all solvent exposed. The most significant difference in the distribution of these charged residues is that they are primarily clustered in one region in NCR13_PFV2, while in NCR13_PFV1, they are generally separated into two regions by a row of hydrophobic (F27, F13, and A14) or neutral (C28) amino acids. Perhaps this different distribution in charge contributes to the different antifungal properties of the two peptides. (TIF) [file ppat.1012745.s007.tif]

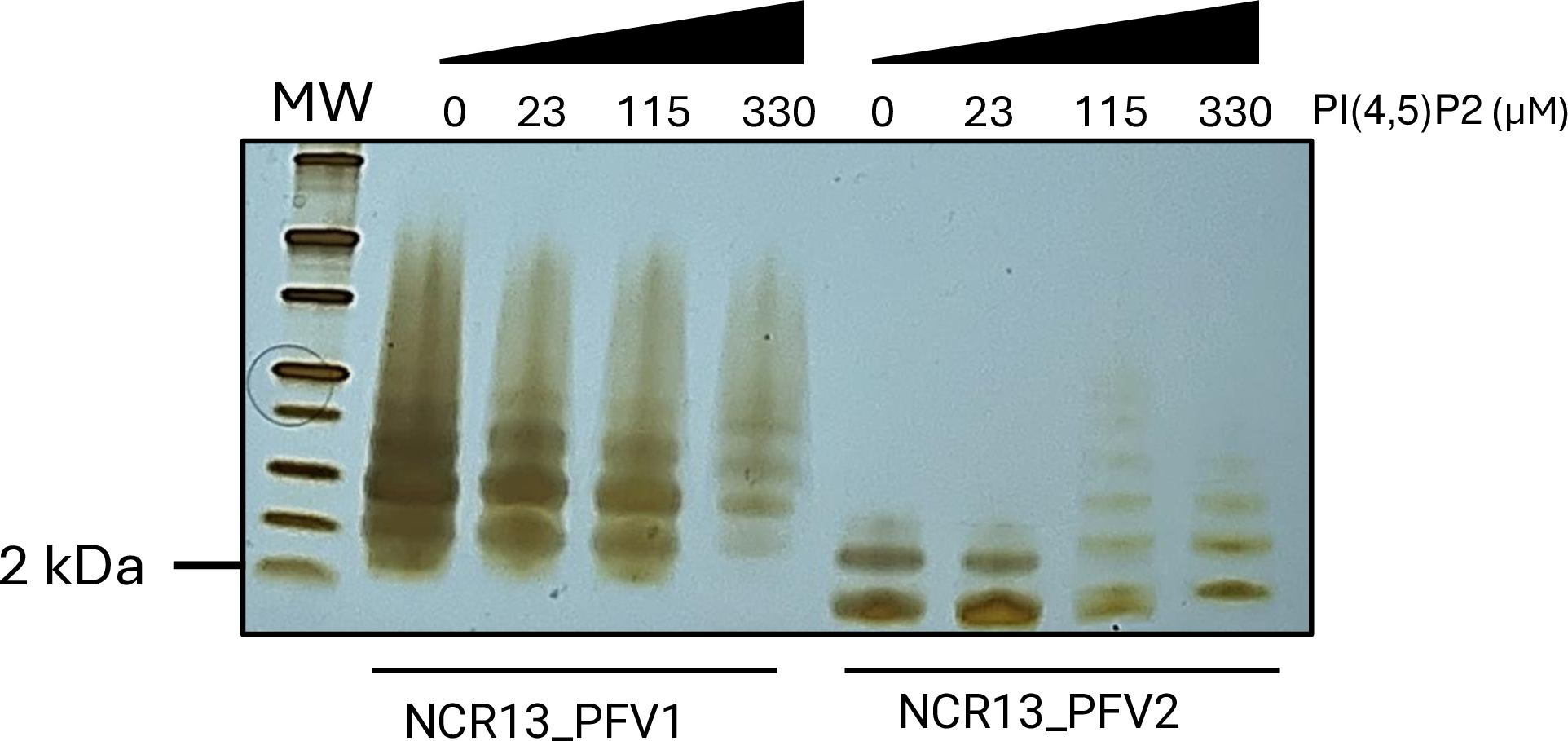

Supplement: S8 Fig — To determine if the peptide oligomerized in the presence of lipid, chemical crosslinking experiments were performed with NCR13_PFV1 and NCR13_PFV2 in the presence of different concentrations of PI(4,5)P2. Following incubation with the biochemical cross-linker bis(sulfosuccinimidyl)suberate (BS3), the peptide was run on an SDS-PAGE gel and the product visualized by silver staining. MW indicates the lane with molecular weight protein marker. The shown image is representative of three independent experiments. (TIF) [file ppat.1012745.s008.tif]

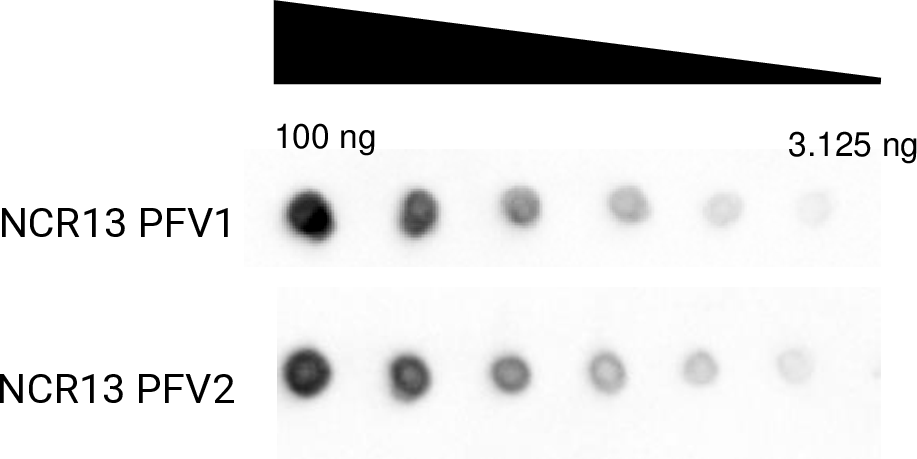

Supplement: S9 Fig — Dot blot analysis of purified NCR13_PFV1 and PFV2 using anti-NCR13 antibody (0.1 μg/mL) followed by goat anti-rabbit IgG HRP (Cytiva RPN4301) at 1:20,000 dilution. (TIF) [file ppat.1012745.s009.tif]

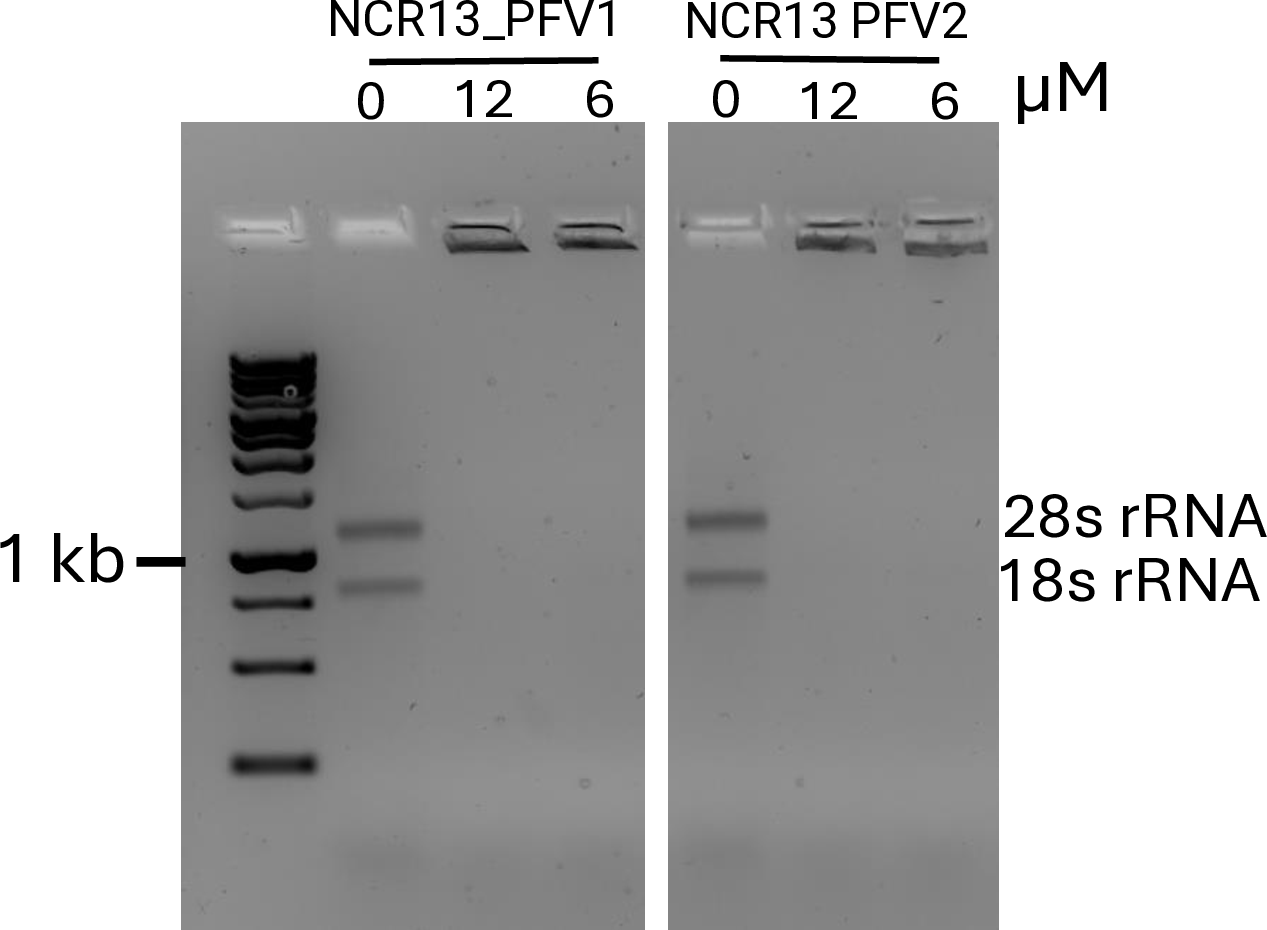

Supplement: S10 Fig — Electrophoretic mobility shift assay (EMSA) to determine if NCR13_PFV1 and NCR13_PFV2 bind rRNA. B. cinerea 28s and 18s rRNA was used to assess binding by electrophoresis on an agarose gel (1%). Peptide concentrations are indicated above the lanes. The first lane on the left contains molecular weight markers. (TIF) [file ppat.1012745.s010.tif]

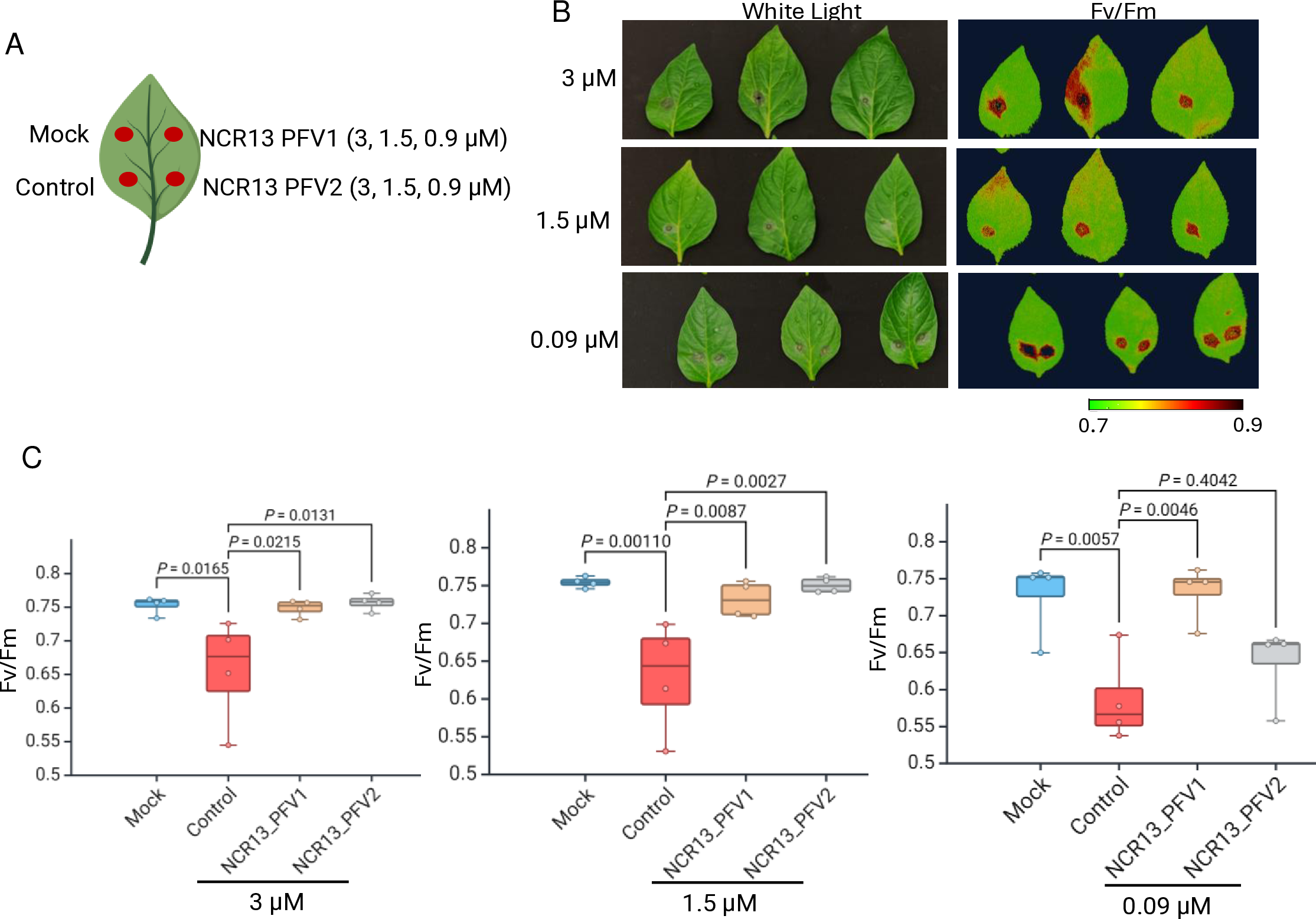

Supplement: S11 Fig — Semi-in planta antifungal activity of NCR13_PFV1 and NCR13_PFV2 against B. cinerea on detached pepper leaves (A) Model of each treatment location on the pepper leaf (B) Representative pictures (under white light and with CropReporter) showing the antifungal activity of NCR13_PFV1 and NCR13_PFV2 at 3, 1.5, and 0.09 μM against B. cinerea on detached pepper leaves. N = 4, N refers to biological replicates. (C) Photosynthetic efficiency (Fv/Fm) measurements of diseased lesions. In the box plot, horizontal lines represent the median and boxes indicate the 25th and 75th percentiles. Statistical significance between control and treated samples was tested using One way ANOVA with Dunnett Multiple comparison test. (TIF) [file ppat.1012745.s011.tif]

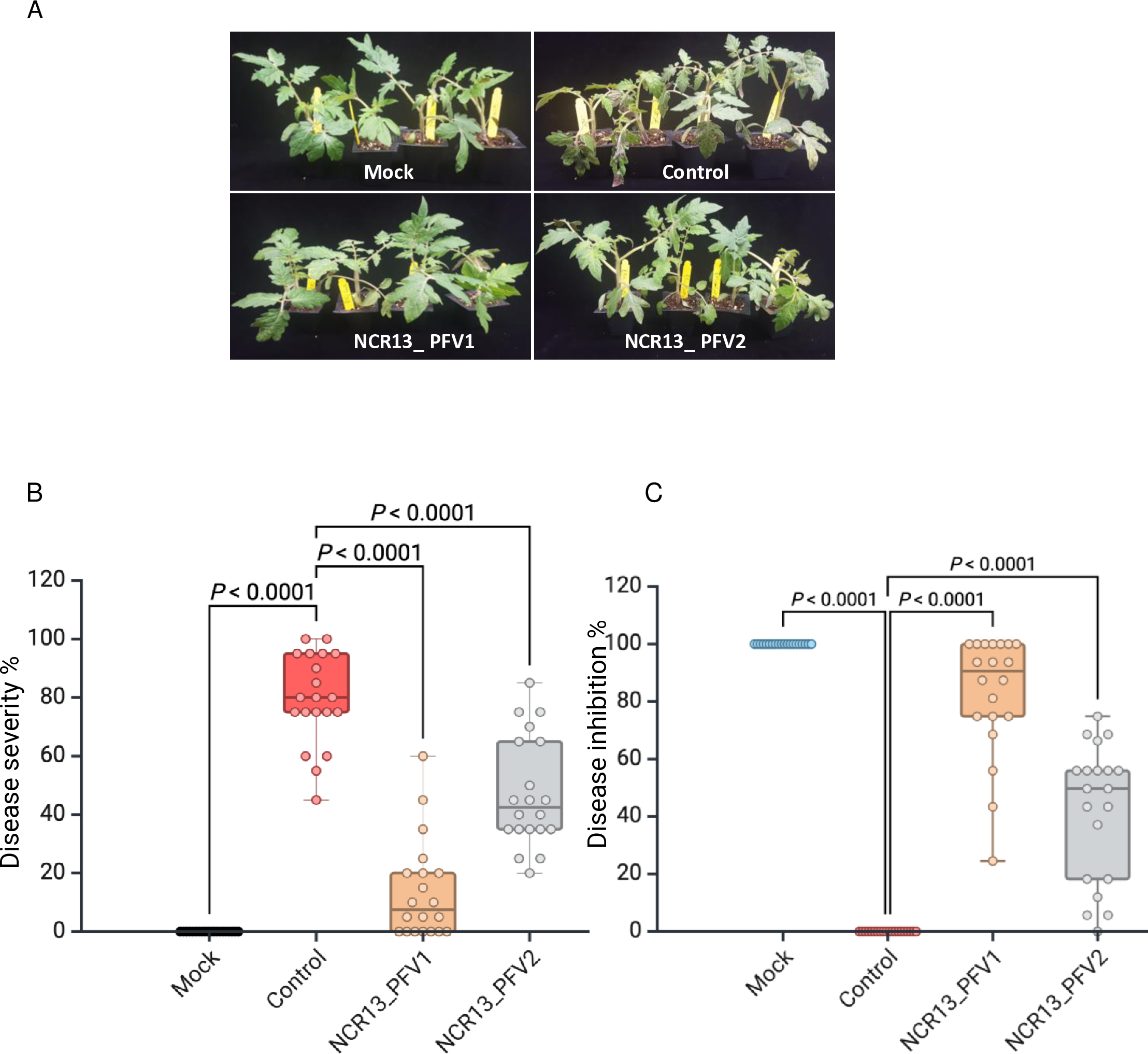

Supplement: S12 Fig — Four-weeks old tomato plants sprayed with 1 mL of a 5 × 10^4^ B. cinerea spore suspension followed 24 h later by a spray containing either 2 mL of water or NCR13_PV1 or NCR13_PV2 at 3 μM. (A) Representative pictures showing the curative antifungal activity of NCR13_PFV1 and NCR13_PFV2 at 3 μM against B. cinerea on tomato leaves. (B) Disease severity %; (C) Disease inhibition %. For panel (C) and (D) each data point represent mean ± SEM denoted by a dot. The average of 5 leaves per plant for 4 plants per treatment. Statistical significance between control and treated samples were tested using One way ANOVA with a Dunnett Multiple comparison test. Three independent experiments were conducted with similar results. (TIF) [file ppat.1012745.s012.tif]
