# Supplementary material for: Chickpea NCR13 disulfide cross-linking variants exhibit profound differences in antifungal activity and modes of action
Source: PLoS Pathog. 2024 Dec 2;20(12):e1012745. doi: 10.1371/journal.ppat.1012745 (PMC11637438; doi:10.1371/journal.ppat.1012745)
Supplement: S1 Table — (PDF) [file ppat.1012745.s013.pdf]

Table S1. Yields of NCR13 from HPLC peaks 1 and 2 obtained from various purification batches of from *Pichia pastoris*

Batch No.	NCR13_peak 1<br>mg/mL	NCR13_peak 2<br>mg/mL
1	2.3	9.3
2	5.3	22.8
3	6.7	23.4
4	9.9	11.0
5	13.4	26.9
6	7.0	17.5
