# Supplementary material for: Chickpea NCR13 disulfide cross-linking variants exhibit profound differences in antifungal activity and modes of action
Source: PLoS Pathog. 2024 Dec 2;20(12):e1012745. doi: 10.1371/journal.ppat.1012745 (PMC11637438; doi:10.1371/journal.ppat.1012745)
Supplement: S2 Table — (PDF) [file ppat.1012745.s014.pdf]

Table S2. Chemical shifts (ppm) for the $^{13}C^{b}$ carbons of the six cysteine residues in oxidized NCR13_PFV1 and NCR13_PFV2.

	C4	C10	C15	C23	C28	C30
**NCR13_PFV1**	47.4	38.8	41.5	39.1	37.2	39.5
**NCR13_PFV2**	42.7	40.9	41.1	47.8	46.1	40.0
**|PFV1-PFV2|**	4.7	2.1	0.4	8.7	8.9	0.5
