# Supplementary material for: Chickpea NCR13 disulfide cross-linking variants exhibit profound differences in antifungal activity and modes of action
Source: PLoS Pathog. 2024 Dec 2;20(12):e1012745. doi: 10.1371/journal.ppat.1012745 (PMC11637438; doi:10.1371/journal.ppat.1012745)
Supplement: S3 Table — (PDF) [file ppat.1012745.s015.pdf]

Table S3. Summary of the structural statistics for the solution structures of NCR13_PFV1 and NCR13_PFV2.

Peptide Name	NCR13_PFV1	NCR13_PFV2
PDB ID	8ULM	7TH8
BMRB ID	31111	30979
**Restraints for Structure Calculations**		
Total NOEs	316	119
Intraresidue NOEs	98	41
Sequential (i, i + 1) NOEs	107	42
Medium-range (i, i + j; 1 < j ≤ 4) NOEs	34	4
Long-range (i, i + j; j > 4) NOEs	54	11
Phi (Φ) angle restraints (2 restraints/angle)	48	50
Psi (Ψ) angle restraints (2 restraints/angle)	48	50
Hydrogen bonds restraints (2 restraints/bond)	12	12
Disulfide bond restraints (3 restraints/bond)	9	9
**Structure Calculations**		
Number of structures calculated	100	100
Number of structures used in ensemble	20	20

**RMSD to Mean (Å)**<br><br>**for PSVS Ordered Residues[b]**		
Backbone N-C$^{\alpha}$-C=O Atoms	0.41 ± 0.13 Å	0.76 ± 0.20 Å
All Heavy Atoms	1.06 ± 0.14 Å	1.54 ± 0.17 Å
**Ramachandran Plot Summary**<br><br>**(from Procheck)**		
Most favored regions	77.7%	85.4%
Additionally allowed regions	22.3%	11.3%
Generously favored regions	0.0%	0.0%
Disallowed	0.0%	0.0%
**Global Quality Scores (Z-score (raw))[c]**		
Procheck (all)	-4.61 (-0.78)	-3.43 (-0.58)
Procheck (Φ, Ψ)	-3.30 (-0.92)	-2.71 (-0.77)
MolProbity clash score	-0.77 (13.40)	-0.66 (12.71)

[a]All statistics are for the ensemble deposited in the Protein Data Bank.

[b]Ordered residues: NCR13_PFV1 K2 – V31; NCR13_PFV2 C4 – K8, C10-V31.
