# Supplementary material for: Chickpea NCR13 disulfide cross-linking variants exhibit profound differences in antifungal activity and modes of action
Source: PLoS Pathog. 2024 Dec 2;20(12):e1012745. doi: 10.1371/journal.ppat.1012745 (PMC11637438; doi:10.1371/journal.ppat.1012745)
Supplement: S4 Table — (PDF) [file ppat.1012745.s016.pdf]

Table S4. Summary of molecular weight of NCR13 disulfide knockout variants determined using mass spectrometry.

Peptide Variant Name	Predicted MW (Daltons) reduced /oxidized	Experimental MW (Daltons)
NCR13_C4S-C23S	3696 / 3692	3692
NCR13_C10S-C28S_P1	3696 / 3692	3692
NCR13_C10S-C28S_P2	3696 / 3692	3692
NCR13_C15S-C30S	3696 / 3692	3692
