# Supplementary material for: Chickpea NCR13 disulfide cross-linking variants exhibit profound differences in antifungal activity and modes of action
Source: PLoS Pathog. 2024 Dec 2;20(12):e1012745. doi: 10.1371/journal.ppat.1012745 (PMC11637438; doi:10.1371/journal.ppat.1012745)
Supplement: Attachment — Submitted filename: Response to reviewers.pdf [file ppat.1012745.s017.pdf]

Response to Reviewers

We thank the editor and the two reviewers for the positive comments. In the revised manuscript, we have addressed the conceptual and technical concerns of each reviewer. The detailed point-by-point response to each of the concerns expressed by reviewers is provided below.

Reviewer #1: 1) How do the different structural arrangements adopted by NCR13_PFV1 and NCR13_PFV2 affect the charge distribution on their surfaces and their potential interaction with anionic phospholipids present on fungal membranes? How are amphipathicity or hydrophobicity compare in these structures and to what extend differences in these properties might have affected the results? Please discuss in the manuscript, including possible consequences on the peptides' antifungal activities.

We examined the surface charge distribution of NCR13_PFVs. As expected, the side chains of the 10 positively charged and 2 negatively charged residues are all solvent exposed on the surface of the NCR13_PFVs. In the revised manuscript, we include a new Supplemental Figure S7 illustrating the electrostatic potential on the solvent exposed surfaces of both peptides. There are only modest differences in the charge distribution, with the negatively charged residues all generally clustered in one region in NCR13_PFV2 while a column of hydrophobic residues separates the negatively charged residues into two regions in NCR13_PFV1.

We have added the following sentences to address this comment, lines 396-400: "Both NCR13_PFVs are positively charged and electrostatic interactions with the negatively charged plasma membrane likely are important for binding to membrane phospholipids. The modestly different distribution of these charges on the surface of these NCR13_PFVs, illustrated in Fig S7, may be responsible for the experimentally observed binding differences." However, these experiments are performed on lipid strips and don't necessarily translate to how NCR13_PFVs interact with lipids in the fungal membranes.

How are amphipathicity or hydrophobicity compare in these structures and to what extend differences in these properties might have affected the results?

Lines 240-244: "In both isomers the organization of the elements of secondary structure do not appear to be stabilized by a hydrophobic core as the hydrophobic residues are mostly solvent exposed. Consequently, the overall structure of each PFV is largely governed by the β-sheet and three disulfide bonds. It is not clear how important this observation is with regard to the antifungal activity of these peptides."

We have also made the following addition to the discussion. Lines 641-643: "Both NCR_PFVs bind to the same phospholipids on lipid strips with different binding intensity that perhaps may be related to the modest difference in the distribution of these charges on the peptide's surface."

2) Along the same lines, the authors have utilized alanine scanning variants to determine the sequence motif governing the antifungal activity of NCR13. However, what are the differences in cationicity, amphipathicity and hydrophobicity among these variants and can differences in these properties be the reason behind the loss activity in some variants? The authors should present such changes in protein properties and discuss how their results might have been affected or dictated by these changes.

The alanine scanning mutagenesis was performed using the chemically synthesized peptides in their reduced state without disulfide bonds. Alanine is most commonly used to ascertain the importance of amino acid residues within a peptide since it eliminates side chain interactions without altering the main chain conformation. The major effect of substituting alanines in the primary sequence of NCR13 is the reduction in the net charge and/or increase in the hydrophobicity of each variant. In the Fig 6A, we have added the net charge of these variants. We found that reduction in the net positive charge correlates with a loss of antifungal activity in NCR13_Ala2, NCR13_Ala3 and NCR13_Ala4). This suggests that net positive charge of the peptide has a major influence on its antifungal activity. The loss of antifungal activity of the NCR13_Ala3 variant was further confirmed by producing this peptide in *Pichia pastoris* in its oxidized form with disulfide bonds formed (Fig. S6B). We expect that in the oxidized form, there will be a significant reduction in the solvent-exposed cationic residues on the surface of the peptide.

We made the changes in the results section, Lines 341-348: "To identify the primary amino acid sequence motif governing the antifungal activity of NCR13 against *B.*

cinerea, we purchased chemically synthesized alanine scanning variants NCR13_AlaV1 through NCR13_Ala5 (Fig 6A). These variants contained a string of three to five consecutive alanine (Ala) residues substituted throughout the NCR13 sequence with altered net charge of the parent primary amino acid sequence. We then compared their antifungal activity relative to NCR13_CS. All experiments were conducted in the reduced state of the peptides lacking disulfide bonds".

3) To what extent is the antifungal activity of NCR13 variants charge-driven and determined by electrostatic attraction with the negatively charged fungal membrane? How is the activity of the peptides affected by increasing salt concentrations? What is the salt concentration the experiments have been performed? Please discuss and if possible, test as well.

The net charge plays an important role in the antifungal activity of NCR13 since NCR13_Ala3 variant with a net charge of +6 has much reduced antifungal activity against *B. cinerea*. The electrostatic interaction also plays a critical role in the antifungal activity of NCR13_PFVs since we observed that NCR13_PFVs become inactive (MIC >12 μM) in the presence of 100 mM NaCl. The 13-residue active motif of NCR13 peptide identified by alanine scanning mutagenesis also loses antifungal activity two-fold. The table below presents the MIC values of NCR13_PFVs in absence and presence of NaCl.

**Peptide**	**Sequence**	**MIC**	
		**SFM (μM)**	**SFM+100 mM NaCl (μM)**
NCR13_PFV1 or PFV2	ATKPCQSDKDCKKFACRKP KVPKCINGFCKCVR	PFV1 (0.09) or PFV2 (1.5)	Inactive (>12)
NCR13 active motif	KKFACRKPKVPKC	3	6
SFM= Synthetic Fungal Medium			

All the experiments were conducted in low-salt synthetic fungal medium (SFM) as per the salt concentration recipe detailed by (Liang et al., 2001).

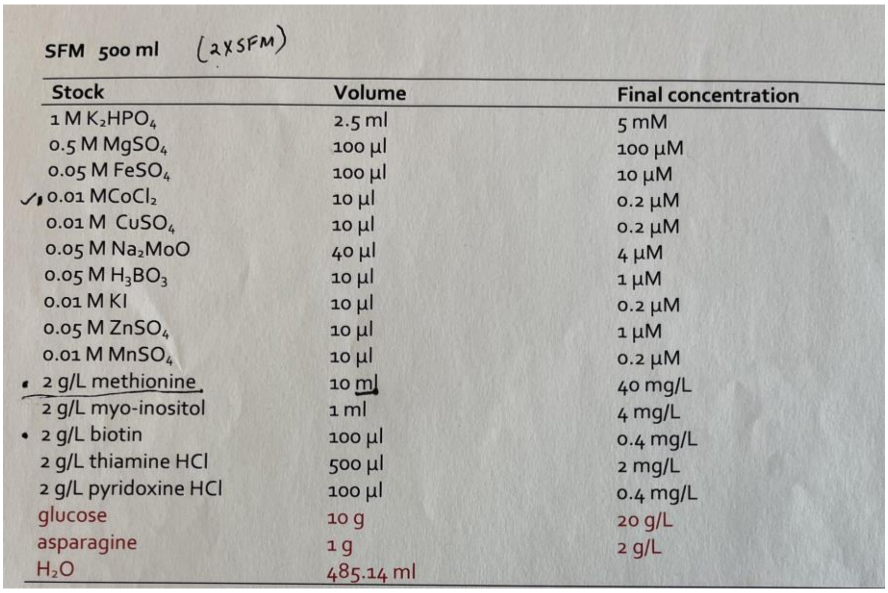

SFM 500 ml (2xSFM)

Stock	Volume	Final concentration
1 M K_2HPO_4	2.5 ml	5 mM
0.5 M $MgSO_4$	100 µl	100 µM
0.05 M $FeSO_4$	100 µl	10 µM
0.01 M$CoCl_2$	10 µl	0.2 µM
0.01 M $CuSO_4$	10 µl	0.2 µM
0.05 M Na_2MoO	40 µl	4 µM
0.05 M H_3BO_3	10 µl	1 µM
0.01 M KI	10 µl	0.2 µM
0.05 M $ZnSO_4$	10 µl	1 µM
0.01 M $MnSO_4$	10 µl	0.2 µM
2 g/L methionine	10 ml	40 mg/L
2 g/L myo-inositol	1 ml	4 mg/L
2 g/L biotin	100 µl	0.4 mg/L
2 g/L thiamine HCl	500 µl	2 mg/L
2 g/L pyridoxine HCl	100 µl	0.4 mg/L
glucose	10 g	20 g/L
asparagine	1 g	2 g/L
H_2O	485.14 ml	

4) The NCR13 construct with all six Cys residues replaced with Ser (NSR13) has no activity against *B. cinerea*, which in part has led the authors to conclude that disulfide bonds are essential to the biological activity of NCR13 (lines 318-322). However, the chemically synthesized NCR13 which lacks disulfide bonds as well has a MIC of 3 uM against B. cinerea (line 279). Why this difference between the two isoforms?

The difference in antifungal activity between the NSR13 construct and the chemically synthesized NCR13_CS likely results from the presence of cysteine residues in NCR13_CS, even though it lacks disulfide bonds. The chemically synthesized NCR13_CS retains antifungal activity with an MIC of 3 µM, which suggests that while disulfide bonds enhance NCR13's activity and they are not strictly necessary for antifungal activity *in vitro*. However, our data indicate that replacing all cysteines with serines, as in NSR13, completely abolishes activity, implying that cysteine residues themselves (even without disulfide bonding) are necessary for any antifungal function. We have revised the manuscript (see lines 326-328) stating that "cysteine residues are essential for NCR13's antifungal activity with disulfide bonds being essential for realizing its full antifungal potential".

5) Functional analysis of the disulfide-bonds in NCR13 by means of site-directed Cys to Ser mutagenesis led the authors to conclude that "...the disulfide pairing of C4-C23 and C15-C30 is essential for the formation of NCR13_PFVs" (lines 310-311). However, NCR13-PVF2 lacks the C4-C23 and it is still functional. Please modify or further clarify your conclusion statement.

Our findings indicate that the disulfide pairing of C4-C23 and C15-C30 plays an important role in forming distinct NCR13_PFVs, though we did not specifically analyze the disulfide bonding pattern in these variants. We acknowledge that alternative disulfide arrangements can also yield functional PFVs, as demonstrated by the activity of NCR13_PFV2.

We have further clarified these statements in the manuscript lines 314-316: "disulfide pairing of C4-C23 and C15-C30 plays an important role in formation of distinct NCR13_PFVs. Nevertheless, we recognize that alternate disulfide arrangements can also yield functional forms, as observed with NCR13_PFV2". Lines 311-312: "....into two presumed disulfide cross-linking variants each containing two disulfide bonds. However, the disulfide pairing of the four Cys residues in these variants remains to be determined"

6) The authors observed that NCR13_PFV1 can permeabilize the plasma membrane of *B. cinerea* faster and at lower concentrations as compared to NCR13_PFV2. An explanation given by the authors is that NCR13_PFV1 adopts a more compact which enables it to cross more readily the fungal cell wall and membrane (lines 633-635). This argument assumes that the two variants are utilizing the same mechanism to cross the membranes and enter the cytosol, but what are the indications for that? Given their localization in different cellular foci, can it be that one, or both but at different degrees depending on properties and peptide concentration, utilizes an energy-dependent endocytic pathway as opposed to an energy independent mechanism? Please discuss in the manuscript.

We have addressed this issue in the manuscript (lines 650-654). "It is plausible that more compact folding of NCR13_PFV1 allows it to more quickly penetrate the cell wall and pass through the outer and inner leaflets of the plasma membrane. However, the possibility remains that these two peptides use different mechanisms to cross the fungal cell wall and disrupt the plasma membrane. Further studies are needed to determine if these two peptides use different energy-dependent or -independent and concentration-dependent pathways for entry into fungal cells. It also remains to be determined if the entry of each peptide into fungal cells requires endocytosis".

Reviewer #2: None. I would like to see some additional data (please see the next section), but they are not major issues.

Part III – Minor Issues: Editorial and Data Presentation Modifications

Please use this section for editorial suggestions as well as relatively minor modifications of existing data that would enhance clarity.

Reviewer #1: Line 35: It is indicated in the Abstract that the net charge of NCR13 is +9, but in Results is reported as +8. Which one is correct?

The net charge is +8. We have corrected it.

Line 48: change to "...a structural framework for designing..."

Done

Line 57: add 'of' prior to 'legume'.

Done

Line 79: add 'the' prior to 'evolution'.

Done

Line 122: it is mentioned that "NCR13 disulfide variants displayed notable differences in fungal cell entry, cell permeabilization,..". is there a difference between the two? (cell entry and cell permeabilization).

Cell permeabilization should be plasma membrane permeabilization. This has been corrected (Line 123) "...notable differences in timing of fungal cell entry and plasma membrane permeabilization"

Reviewer #2: 1. It is believed that NCR peptides evolved to interact with rhizobia. Could the authors speculate why NCR13 has such high activities against several fungi? Is NCR13 special in that it has evolved to target fungi? Or does it have functions similar to other NCR peptides in the legume-rhizobia symbiosis, but such activities happen to be potent on fungi as well?

While NCR peptides are primarily known for their roles in legume-rhizobia symbiosis, NCR13's high broad-spectrum antifungal activity against several fungal pathogens likely results from its structure, high net charge, sequence composition as well as distribution of the cationic and hydrophobic residues on the surface of the peptide. Currently, role of NCR13 in chickpea-rhizobia symbiosis remains unclear. To elucidate the role of NCR13

during symbiosis, it will be necessary to generate CRISPR knockout of the NCR13 gene in chickpea and determine the role of NCR13 on bacteroid differentiation and nitrogen fixation.

2. Related to the above question, is the behavior of NCR13_PFV1 observed in this study relevant to its function in the legume-rhizobia symbiosis? Specifically, the authors showed that this peptide localizes to the nucleoli, binds rRNA, and inhibits translation in fungi. The bacterial counterparts are either very different, or missing. Do the authors believe NCR13 interferes with protein translation in rhizobia also? If so, does it act through a conserved mechanism?

While *Pichia pastoris* generated two disulfide cross-linking variants of NCR13, there is no guarantee that chickpea nodules also generate these two variants. In our study, we showed that NCR13_PFV1 localizes to the nucleoli, binds rRNA, and inhibits translation. It will be very informative to determine the role of this peptide during chickpea-*Mesorhizobium ciceri* symbiosis. In particular, it will be important to determine if this peptide permeabilizes rhizobial cells, gains entry into the cytoplasm of bacterial cells and inhibits protein synthesis. Considering the peptide's potent antimicrobial activity, it is likely that it may interact with bacterial cellular components differently to modulate protein translation or other physiological processes relevant to symbiosis. Future investigation would be essential to examine NCR13's interaction with rhizobial cells.

3. Can the authors speculate which form is present in the nodule, or do they believe NCR13 exists in more than one-fold, as observed in Pichia?

The native conformation(s) of NCR13 *in planta* remains unknown due to insufficient quantities in root nodules for purification and analysis. However, the potential existence of NCR13 disulfide variants with distinct folding patterns *in planta* cannot be overlooked. We believe this manuscript would create interest in the research community to look at NCR13 disulfide variants or any NCR peptide variants *in planta*.

4. Still related to the first question, does any of the three forms of NCR13 (PFV1, PFV2, and chemically synthesized) show activities against rhizobia? If so, do they have even lower MICs when tested against rhizobia?

Since the focus of the studies in our lab is on testing the antifungal properties of NCR peptides, we did not test the activity of the NCR13 variants (PFV1, PFV2, or the chemically synthesized NCR13) against rhizobia. We understand that this is an important question to address. While it is plausible that these peptides might inhibit *M. ciceri* at certain concentrations, it will be necessary to conduct antibacterial assays of NCR13 peptides using this bacterial endosymbiont.

5. If the peptide is highly toxic to three very different pathogenic fungi, how could the authors produce this peptide in Pichia, another fungal species? Is it toxic to Pichia, and did the author have to adjust the induction condition so as to be able to isolate the peptide before the yeast cells died?

In our lab, several cationic peptides including NCR peptides and defensins with potent antifungal activity have been successfully expressed in *P. pastoris*. NCR13 is no exception. Under the peptide expression conditions described in the manuscript, NCR13 does not exhibit toxicity to *P. pastoris*.

6. Perhaps related to the above question, the authors noted that the PFV1 form, which is much more active, consistently yielded less than PFV2. Is it because the PFV1 form is more toxic to Pichia and thus cannot be produced in large quantities?

It is indeed possible that the PFV1 yields less than PFV2 because *P. pastoris* cells can tolerate only a threshold level of the PFV1 form. We can speculate that the higher activity of PFV1 might cause stress responses in *P. pastoris* cells, thus limiting its expression. Other factors such as differences in the disulfide cross-linking efficiency, stability, or secretion pathways between PFV1 and PFV2 could also impact peptide folding variant yield.

7. The chemically synthesized NCR13 has a retention time different from either PFV1 or PFV2. How do the authors think it is folded? Is it reduced? Or is it simply misfolded? Is it a cautionary tale for studies that use chemically synthesized NCR peptides in general?

We confirm that NCR13_CS was purchased in a reduced form, and mass spectrometry analysis validated that it predominantly remains in the reduced state. The difference in

retention time that we observed for NCR13_CS compared to PFV1 and PFV2 may reflect a more linear or unfolded conformation due to the absence of disulfide linkages. As highlighted in lines 610-612, the reduced form of NCR13_CS showed approximately a 33-fold decrease in antifungal activity compared to biosynthetically produced NCR13_PFV1 and a 2-fold decrease compared to NCR13_PFV2. This observation is consistent with findings from our previous study on NCR044, where the *Pichia*-synthesized NCR044 showed a significantly higher antifungal activity with IC_{50} of 1.55 ± 0.21 μM, while the chemically synthesized reduced form had an IC_{50} of 4.16 ± 0.18 μM (Velivelli et al., 2020). These results highlight the importance of peptide folding in accurately interpreting the biological activity of chemically synthesized NCR peptides, as reduced forms may not reflect their bioactive states. We agree with the reviewer that it is necessary to exercise caution using chemically synthesized NCR peptides in general.

Liang, J., Shah, D. M., Wu, Y., Rosenberger, C. A., & Hakimi, S. M. (2001). *Antifungal polypeptide from alfalfa and methods for controlling plant pathogenic fungi* (USA Patent No. US 6316407).

Velivelli, S. L. S., Czymmek, K. J., Li, H., Shaw, J. B., Buchko, G. W., & Shah, D. M. (2020). Antifungal symbiotic peptide NCR044 exhibits unique structure and multifaceted mechanisms of action that confer plant protection. *Proc Natl Acad Sci U S A*, *117*(27), 16043-16054. https://doi.org/10.1073/pnas.2003526117
